# Platypnea-Orthodeoxia: An Unusual Case of Hypoxemia

**DOI:** 10.1155/2011/104653

**Published:** 2011-07-24

**Authors:** Jennifer Slim, Jennifer McNear, Rachel Beck, Robert Saad, Jorge Alvarez, Ahmad Slim

**Affiliations:** ^1^Cardiology Service, Brooke Army Medical Center, 3851 Roger Brooke Drive, Fort Sam Houston, TX 78234-6200, USA; ^2^Cardiology Service, Cardiology Clinics of San Antonio, 4411 Medical Dr, Suite #300, San Antonio, TX 78229-3824, USA

## Abstract

A 52 year old female presented for two weeks of acute onset dyspnea on exertion. She was found to be hypoxic with a room air saturation of 88%. Baseline echocardiogram was normal with the exception of aortic root dilation. Right and left heart catheterizations were performed. The coronary arteries were normal in original and without disease. The right heart catheterization demonstrated normal pulmonary pressures and “no evidence of intra-cardiac shunt”. Repeat echocardiogram was performed with agitated saline contrast and revealed a small amount of right to left shunting across the intra-atrial septum with cough while supine and significant right to left shunting while upright; these findings were consistent with the presence of a patent foramen ovale (PFO) and platypnea-orthodeoxia syndrome. The patient underwent percutaneous closure of her PFO with an Amplatzer device, and exhibited rapid resolution of her symptoms and hypoxia. She is off oxygen and has returned to work as a nurse practitioner. The case highlights the importance of clinical vigilance and consideration of this syndrome in the differential diagnosis of unexplained hypoxia. Our patient had a dramatic and positive outcome: complete alleviation of dyspnea and oxygen dependence after PFO closure.

## 1. Case Report

A 52-year-old female presented for two weeks of acute onset dyspnea on exertion. She was found to be hypoxic with a supine room air saturation of 88%. She underwent extensive evaluation by numerous specialists throughout Texas without a diagnosis. 

Routine chemistry, coagulation panel, and CBC were unremarkable. A D-dimer was negative and BNP was 20. Chest X-ray was unremarkable aside from hiatal hernia. ECG was sinus rhythm with nonspecific ST changes. Patient underwent CT of the chest which was negative for pulmonary embolism but showed dilation of the ascending aorta to 4.6 cm. Baseline echocardiogram was normal with the exception of aortic root dilation. Right and left heart catheterizations were performed. The coronary arteries were normal in original and without disease. The right heart catheterization demonstrated normal pulmonary pressures without step up in oxygen saturation suggestive of intracardiac shunt. 

Given persistence of symptoms, limited exercise tolerance, and need for supplemental oxygen, the patient was referred for a second opinion. Repeat echocardiogram was performed with agitated saline contrast and revealed a small amount of right to left shunting across the intra-atrial septum with cough while supine and significant right to left shunting while upright; these findings were consistent with the presence of a patent foramen ovale (PFO) and platypnea-orthodeoxia syndrome. 

The patient underwent percutaneous closure of her patent foramen ovale with an Amplatzer device and exhibited rapid resolution of her symptoms and hypoxia. She is off oxygen and has returned to work as a nurse practitioner.

## 2. Discussion

Platypnea-orthodeoxia syndrome is an uncommon disorder characterized by dyspnea and arterial desaturation in the upright position with improvement in the supine position. The development of this syndrome requires two components: a shunt (interatrial or intrapulmonary) and a functional component that promotes abnormal shunting when the patient moves from a supine to an upright position.

Platypnea-orthodeoxia syndrome is likely an under-recognized cause for dyspnea and positional hypoxia. Our patient had both required features for the syndrome, intra-atrial shunt and a functional component that promotes shunting. She had a patent foramen ovale as well as aortic root dilation. Dilation of the aortic root typically causes the intra-atrial septum to become more horizontal. This horizontal intra-atrial septum aligned the patent foramen ovale with inferior vena caval inflow, the “perfect storm” to provoke shunting while upright. In an autopsy study of 965 subjects, probe-patent PFO was present in 27 percent of subjects with equal distribution among gender. The size of the PFO was 1 to 10 mm in diameter in 98% of the autopsied hearts [1]. The prevalence of PFO is increased in patients with cryptogenic CVA (<age 55). In one study of patients with cryptogenic CVA (mean age of 42), a PFO was present in 37% of the subjects and a PFO with an atrial septal aneurysm (ASA) was detected in 9% [2]. PFOs are usually identified with an agitated saline contrast study augmented by cough or Valsalva maneuver. Both are thought to open the PFO via increased right atrial filling. In a small study using transesophageal echocardiography, the detection rate improved from 57% to 92% with Valsalva [3]. However, positional shunting may be overlooked without upright provocative testing.

The platypnea-orthodeoxia syndrome is a rare condition with paradoxical dyspnea (platypnea) and arterial desaturation when patient is upright and resolution of symptoms with improvement in saturation when patient is supine (orthodeoxia) [4]. The mechanism proposed depends on the presence of a shunt (intra-atrial or pulmonary) and a condition that increases shunting from right to left in the upright position usually initiated by an increase in right atrial pressure (i.e., ASA, right atrial deformity, etc.). Some of the clinical entities that are known to be associated with platypnea-orthodeoxia include dilated aorta, persistent eustachian valve, constrictive pericarditis, pulmomary disorders (emphysema, arteriovenous malformation, pulmonary embolism), gastrointestinal, as well as neural (autonomic dysfunction) [5]. Some authors advocate measuring oxygen saturation in the supine and standing position when suspected and even the use of tilt-table testing [6]. The definitive therapy is closure of the defect with an approved closure device.

## 3. Conclusion

There are several case reports in the literature of platypnea-orthodeoxia syndrome in the setting of patent foramen ovale and aortic root dilation. The case highlights the importance of clinical vigilance and consideration of this syndrome in the differential diagnosis of unexplained hypoxia. Our patient had a dramatic and positive outcome: complete alleviation of dyspnea and oxygen dependence after PFO closure.

##  Disclosure

The opinions or assertions contained herein are the private views of the authors and are not to be construed as reflecting the views of the Department of the Army or the Department of Defense.
